# A model of immunohistochemical differences between invasive breast cancers and DCIS lesions tested on a consecutive case series of 1248 patients

**DOI:** 10.1186/1742-4682-11-29

**Published:** 2014-06-11

**Authors:** Sven Kurbel, Ksenija Marjanović, Branko Dmitrović

**Affiliations:** 1 Department of Physiology, Osijek Medical Faculty, Osijek, Croatia; 2 Department of Pathology and Forensic Medicine, Osijek University Hospital, Osijek, Croatia

## Abstract

**Background:**

A previous theoretic model (Tumour Biol 2013;34:1–7.) that breast tumor types differ in the relative rate of tissue invasion was elaborated and developed on a consecutive case series.

**Method:**

Histologic data of 68 ductal breast cancer in situ (DCIS) and 1180 invasive ductal cancer (IDC) patients were collected and analyzed.

**Results:**

ER^+^PgR^−^ phenotype was more common in Luminal B2 than among the pooled Luminal A&B1 (p = 0.0002), and more frequent in Luminal B1 than in Luminal A (p = 0.0167). The same phenotype was associated with the age older than 54 years in Luminal B1 and in B2 patients. HER2 type cancers were more frequent in older patients (p = 0.0038).

Tumor progression from DCIS to IDC was found 39% faster than the average in Luminal B1 tumors, supporting the clinical importance of this tumor type. A rare combination of low Ki-67 in HER2 type cancers (only 14% of HER2 type cancers) showed very slow transition to IDC (occurring at only 53.55% of average progression rate), while triple-negative cancers progressed faster than the average, despite Ki-67 value (104.63% for low and 114.27% for high Ki-67 tumors).

In three tumor types with positive steroid receptors the ER^+^PgR^−^ phenotype showed slower IDC transition than the ER^+^PgR^+^ phenotype of the same tumor type (difference in progression rate was 38% for Luminal A, 46% for Luminal B1 and 67% for Luminal B2 with Ki67 > 14%).

Triple-negative tumors in younger patients exceeded the expected average progression rate by 24%, while in HER2 type tumors, the rate of tissue invasion was in younger patients 20% lower than the expected value.

**Conclusions:**

The relative rate of tissue invasion differed substantialy among our patients. Differences depended on tumor types, steroid expression phenotypes and age. The dysfunctional ERs in the ER^+^PgR^−^ phenotype showed slower rates of tissue invasion, suggesting that ligand binding to functional breast tumor ERs, beside promoting the PgR expression, possibly also promotes tumor transition to the invasive phase.

In triple-negative tumors, an age dependent premenopausal mechanism possibly acted as an accelerator of tissue invasion, while faster tissue invasion by HER2-overexpressed tumors in older patients possibly depended on an unidentified mechanism that takes more time to be acquired, so it was less present in premenopausal patients.

## Introduction

Ductal breast cancer in situ (DCIS) seems to precede invasive ductal cancer (IDC). This idea is based on a high degree of similarity between molecular alterations in DCIS and invasive cancer in the same patient [1,2], although triple-negative invasive cancers may seem almost to lack their triple-negative DCIS precursor [3-6].

If all breast cancer types evolve from DCIS lesions, more aggressive breast cancer types can be recognized by comparing breast tumor type distributions between DCIS lesions and invasive breast cancers [1]. The basic idea is that at the time of breast tumor diagnosis more aggressive tumor types will have fewer DCIS lesions in comparison to less aggressive types with more tumors still in the DCIS phase.

This model, based on reported data of breast cancer characteristics pooled from several studies, was proposed in a recent theoretic article [1]. Differences in the incidences of the main breast cancer types (Luminal A, Luminal B, HER2-overexpressed and the triple-negative tumors) between DCIS and invasive ductal cancers (IDC) were used to calculate the relative rate of progression from the *in situ* stage to invasive form. This calculated value is probably not directly related to the course of disease. Instead, it was designed as a parameter that defines chances of a certain tumor to become invasive at the time of diagnoses. In other words, we were trying to indirectly measure the speed of the critical phase in breast cancer development.

After becoming invasive, the future growth depends on many factors. For instance, HER2-overexpressed tumors were found in the theoretic model to be very slow in tissue invasion [1], although further development of HER2-overexpressed invasive breast cancers, is well known as aggressive and rapid. Triple-negative tumors showed in the pooled model the most rapid rate of tissue progression and this finding was interpreted as a sign of an unrecognized tumor progression mechanism, independent of steroid receptors or HER2 expression.

Further to investigate the idea that immunohistochemical characteristics of IDC and of DCIS tumors from a defined single population can help us to describe biology of breast tumor types, a sufficiently large sample was required of DCIS and IDC patients with defined tumor types, ER, PgR, HER2 status and other histological features. The need for all these data comes from reported breast cancer studies [7-12] that suggested that PgR expression in cells with ER depends on estrogen exposure during previous days. In other words, ligand binding to functional ERs is a physiologic prerequisite for PgR expression in breast tumor cells. This clearly suggests that the three possible positive steroid receptor breast cancer phenotypes (ER^+^PgR^+^, ER^+^PgR^−^ and the rare ER^−^PgR^+^) may be biologically different, particularly in this early phase of tumor growth, during tissue invasion.

Without a suitably detailed published data set from a single population, the only solution was to use a single institution experience in diagnosing 68 DCIS and 1180 IDC patients that has already been attained as a part of an ongoing research project (219-2192382-2426), financed by the Croatian Ministry of Science.

The aim of this study was to apply numeric methods described in the previous theoretic paper [1] on real patient data and thus get more reliable answers on the possible mechanisms underlying the occurrence of breast cancer types.

## Patients, materials and methods

### Patients

Data used for the model testing were taken from the above mentioned ongoing research project (219-2192382-2426), approved by the Ethical Committee of Osijek Medical Faculty as compliant with the Helsinki Declaration, before grant submission to Croatian Ministry of Science and Education.

In this study 1248 consecutive patients with intraductal and/or ductal invasive breast cancers (regardless of stage) were included. All patients were diagnosed and treated in Osijek Clinical Hospital during the time period from January 2004 to December 2012. All specimens were excisional biopsy specimens, or mastectomy specimens. 68 cases were DCIS alone. Tumor grade was determined using the Bloom and Richardson grading scheme [13].

### Immunohistochemistry

Each immunostained slide was evaluated for the presence of ER and PgR expression, HER2 protein overexpression, and Ki-67 proliferation activity. Immunohistochemical staining was done by standard avidin-biotin method (DAKO LSAB®2 System, HRP) using 4 μm sections from representative paraffin blocks. Nuclear staining with anti-ER, PgR, Ki-67 antibodies was done and the percentage of positive cells per 500 tumor cells was calculated. Importantly, all ER^+^ and PgR^+^ cases showed staining in at least 1% of the DCIS and/or invasive tumor cell nuclei, whereas all ER-negative and PgR-negative cases showed complete absence of tumor cell staining (but with staining of normal breast epithelial cell nuclei) [14].

Tumor cells were considered positive for HER2 protein overexpression when more than 10% of the cells showed strong membrane staining (equivalent to a score of 3+ in the DakoCytomation HercepTest). HER2 2+ result was only positive if confirmed by chromogene *in situ* hybridization for gene amplification. All immunostains were initially reviewed and scored by a single pathologist. Hormone receptors were then reviewed and accepted as negative if 100% of cells lacked nuclear immunostaining for hormone receptors.

According to immunohistochemical features, tumors (both DCIS and invasive cancers) were divided into the following five groups: Luminal A (ER^+^ and/or PgR^+^, HER2-negative, Ki-67 < =14%), Luminal B1 (ER^+^ and/or PgR^+^, HER2-negative, Ki-67 > 14%), Luminal B2 (ER^+^ and/or PgR^+^, HER2-positive, any Ki-67), HER2 (ER^−^, PgR^−^, HER2-positive), and triple-negative (ER^−^, PgR^−^, HER2-negative) [15].

### Statistical analysis

Collected data were organized in 2×2 tables and differences from expected frequencies were checked by χ^2^ tests.

The relative rate of tissue invasion was calculated from a simple contingency table of DCIS and IDC data according to tumor types. The probability of tumor progression (p) at the time of diagnosis for each tumor type was calculated using the following equation:

$$p=\frac{\mathrm{BC}}{\mathrm{DCIS}+\mathrm{BC}}$$

where *BC* stands for the number of all invasive BCs for that tumor type, while *DCIS* is the reported number of DCIS lesions of that type.

These results were used to calculate the number of progression t_1/2_ intervals spent prior the time of diagnosis using the following equation:

$$t_{1/2}=\frac{\log\left(1-p\right)}{\log\left(1/2\right)}$$

where *p* is the calculated probability of progression for that tumor type.

This interval is defined as a time required for 50% of DCIS lesions to become invasive. It was then converted into the relative rate (*v*_*rel*_) using the following equation:

$$v_{\mathrm{rel}}=\frac{\mathrm{tumor}\_\mathrm{type}\_t_{1/2}}{\mathrm{average}\_t_{1/2}}$$

where *tumor*_*type*_*t*_*1*/*2*_ is the number of elapsed t_1/2_ for a certain tumor type and *average*_*t*_*1*/*2*_ is the number of elapsed intervals for all included breast tumors.

The model predictions from the ref. 1 were recalculated for the modeled population with only 5% of DCIS at the time of diagnosis. This recalculation with a similar share of DCIS tumors allowed comparison between the model results and here presented data of our patients.

## Results

Table 1 shows distributions of our patients regarding breast tumor type, ER & PgR expression, HER2 overexpression and age (younger than 55, or older than 54). Since the estrogen-dependent PgR expression occurs only in cells with functional ERs, the ER^+^PgR^−^ tumors are considered expressing “dysfunctional” ERs.

**Table 1:** Distributions of patients regarding breast tumor type, ER and PgR expression, HER2 overexpression and age (younger than 55, or older than 54)

	**Luminal A and B1**	**Luminal B2**	**Total**	**Age**	**Luminal A and B1**	**Luminal B2**	**Total**
Distribution of breast cancer patients with positive receptors according to HER2 and age							
ER^+^PgR^+^	666	172	838	<55	218	69	287
ER^+^PgR^−^	66	38	104	>54	525	141	666
ER^−^PgR^+^	11	0	11	Total	743	210	953
Total	743	210	953				
Χ^2^ (p)	13.70 (0.0002)			Χ^2^(p)	2.34 (0.1260)		
Distribution of ductal breast cancer patients according to their steroid receptor phenotype and Ki-67 value							
ER^+^ tumors without HER2	Luminal A (Ki-67 < =14%)	Luminal B1 (Ki-67 > 14%)	Total	Luminal B2	Ki-67 < =14%	Ki-67 > 14%	Total
ER^+^PgR^+^	365	301	666	ER^+^PgR^+^	49	123	172
ER^+^PgR^−^	26	40	66	ER^+^PgR^−^	9	29	38
ER^−^PgR^+^	6	5	11	ER^−^PgR^+^	0	0	0
Total	397	346	743	Total	58	152	210
Χ^2^(p)	5.73 (0.0167)			Χ^2^ (p)	0.36 (0.5489)		
Distribution of ductal breast cancer patients according to their age and other features							
Age of Luminal A patients	ER^+^PgR^+^	ER^+^PgR^−^	ER^−^PgR^+^	Age of HER2 patients	Ki-67 < =14%	Ki-67 > 14%	Total
<55	99	5	4	<55	3	38	41
>54	266	21	2	>54	16	78	94
Total	365	26	6	Total	19	116	135
Χ^2^(p)	0.77 (0.3789)			Χ^2^ (p)	2.22 (0.1360)		
Age of Luminal B1 patients	ER^+^PgR^+^	ER^+^PgR^−^	ER^−^PgR^+^	Age of triple-negative patients	Ki-67 < =14%	Ki-67 > 14%	Total
<55	103	6	1	<55	7	68	75
>54	198	34	4	>54	14	71	85
Total	301	40	5	Total	21	139	160
Χ^2^(p)	6.00 (0.0143)			Χ^2^ (p)	1.78 (0.1822)		
Age of Luminal B2 patients	ER^+^PgR^+^	ER^+^PgR^−^	ER^−^PgR^+^	Age of patients	triple-negative	HER2	Total
<55	65	4	0	<55	75	41	116
>54	107	34	0	>54	85	94	179
Total	172	38	0	Total	160	135	295
Χ^2^(p)	10.49 (0.0012)			Χ^2^ (p)	8.36 (0.0038)		

The ER^+^PgR- phenotype was more common in Luminal B2 in comparison to the pooled Luminal A and B1, in Luminal B1. In Luminal B1 and in B2 ER^+^PgR- tumors were more common in patients older than 54. HER2-overexpressed patients are often older than 54.

Several comparisons were made and they can be summarized in the following statements:

• Dysfunctional ER^+^PgR^−^ phenotype was more common in Luminal B2 (38 out of 210 cases) in comparison to the pooled Luminal A&B1 (only 66 out of 743 cases), suggesting that this combination was more prevalent than expected in our patients (p = 0.0002).

• If Luminal A was compared with B1, high Ki-67 values in B1 are also more often combined with the ER^+^PgR^−^ phenotype (40 out of 346 vs. 26 out of 397 cases, p = 0.0167). Thus, tumors with either HER2 overexpression, or with increased Ki-67 values seemed prone to develop the dysfunctional ER^+^PgR^−^ phenotype.

• Age distributions were similar in the pooled Luminal A&B1 as in B2 (p = 0.1260) suggesting that age and hormone exposure did not matter much in the tumor type differentiation between these two types.

• If Luminal B2 tumors are divided according to Ki-67 value and steroid receptor phenotypes, no important differences were observed (p = 0.5489), suggesting that distribution of steroid phenotypes and Ki-67 values remain unaltered in tumors with HER2 overexpression.

• On the other hand, patients’ age was not important for distribution of steroid receptor phenotypes only in Luminal A patients (p = 0.3789). In Luminal B1 patients 34 out of 40 ER^+^PgR^−^ tumors were detected in patients older than 54 years. Very similar distribution was observed in Luminal B2 patients with 34 out of 38 found in patients older than 54 years. A possible conclusion is that the ER^+^PgR^−^ phenotype is more common in tumors with high Ki-67 values or with HER2 overexpression, particularly in older patients.

• It is interesting that distribution of HER2-overexpressed tumor according to Ki-67 value and patients’ age did not show any difference, and the same was found for triple-negative tumors. Nevertheless, if we compare age of HER2 versus triple-negative patients, HER2 patients are obviously often older than 54 years (94 out of 135 in comparison to 85 out of 160 triple-negative cases, p = 0.0038). This suggests that HER2 tumors might be slower in the initial growth phase so they were more often diagnosed in older patients.

Table 2 shows results of the model of ductal cancer progression from ref. 1, applied to patients in this study. For the comparison purpose only, data from ref. 1. were recalculated to simulate population of breast tumor patients with 5% of DCIS at the time of diagnosis. The recalculation was evidently well matched since the number of spent T_1/2_ of progression was very similar in presented data and in the recalculated simulated population (only above 4 t_1/2_ are spent in both data sets). Relative rates were calculated relative to the average progression rate of all tumors (the average count of spent t_1/2_ for all BC patients was 4.198), making the relative rates easily comparable:

**Table 2:** Results of the ductal cancer progression model from ref. 1 , applied to here presented patients

**Ductal breast cancer (DC) types**		**Ductal breast cancer types**					**Total**
		**Luminal A**	**Luminal B1**	**Luminal B2**	**HER2**^**+**^	**Triple-negative**	
**DCIS**	***A***	**34**	**6**	**12**	**10**	**6**	**68**
**IDC**	***B***	**363**	**340**	**198**	**125**	**154**	**1180**
Number of all DC *(A + B)*	*C*	397	346	210	135	160	1248
% of cancer types in all DC		31.81%	27.72%	16.83%	10.82%	12.82%	100.00%
Probability of progression at the time of diagnosis *(B/C)*	*p*	91.44%	98.27%	94.29%	92.59%	96.25%	94.55%
Number of progression t_1/2_ spent till the time of diagnosis *log(1-p)/log(1/2)*		3.55	5.85	4.13	3.75	4.74	4.20
**Relative rate of progression from DCIS to IDC**		**84.46%**	**139.35%**	**98.36%**	**89.45%**	**112.84%**	**100.00%**
Pooled reported DCIS and IDC data from ref. 1	Type	Luminal A		Luminal B	HER2^+^	Triple-negative	Total
	DCIS	153		53	96	26	328
	IDC	124		68	31	109	332
	Total	277		121	127	135	660
	Simulated population of all ductal breast cancer patients with 5% DCIS	2480.61		1329.43	677.90	2072.05	6560
	% of cancer types in simulated population	37.81%		20.27%	10.33%	31.59%	100.00%
	Probability of progression at the time of diagnosis	93.83%		96.01%	85.84%	98.75%	95.00%
	Number of progression t_1/2_ spent till the time of diagnosis	4.02		4.65	2.82	6.32	4.32

The fastest in tissue invasion were Luminal B1 cancers and then the triple-negative cancers. Based on relative rates of tissue invasion it seems that criteria for Luminal B1 have really identified the most aggressive breast cancers.

• The most rapid progression was observed for Luminal B1 cancers and then for triple-negative cancers. In ref. 1 Luminal B1 cancers were not separated from other Luminal A cancers, thus making triple-negative cancers the fastest in the relative tissue progression rate.

As Luminal B1 cancers differ from Luminal A cancers only in Ki-67 values, the same threshold of 14% Ki-67 value was applied in Table 3 to Luminal B2, HER2-overexpressed and triple-negative cancers:

**Table 3:** Relative tissue invasion rates according to the Ki-67 value

**Binary features**			**DCIS**	**IDC**	**Relative rate**	**Rate difference (positive–negative)**
ER	Negative		16	290	1.0142	−0.0186
	Positive		52	890	0.9955	
	Total		68	1180	1.0000	
PgR	Negative		29	370	0.9010	0.1577
	Positive		39	810	1.0587	
	Total		68	1180	1.0000	
HER2	Negative		46	857	1.0231	−0.0772
	Positive		22	323	0.9459	
	Total		68	1180	1.0000	
Ki-67	<=14%		43	451	0.8390	0.3317
	>14		25	729	1.1707	
	Total		68	1180	1.0000	
Ductal breast cancer types including Luminal B1 and proposed other Ki-67 subtypes	Luminal A		34	363	0.8446	0.5489
	Luminal B1		6	340	1.3936	
	Luminal B2	<=14%	4	54	0.9190	0.0929
		>14%	8	144	1.0119	
	HER2	<=14%	4	15	0.5355	0.4824
		>14%	6	110	1.0179	
	Triple-negative	<=14%	1	20	1.0463	0.0964
		>14%	5	134	1.1427	
	Total		68	1180	1.0000	

Because Luminal B1 cancers differ from Luminal A cancers only by Ki-67 values, the same threshold of the 14% Ki-67 value was applied to Luminal B2, HER2-overexpressed and triple-negative cancers. Luminal B1 phenotype was 39% faster than average in progression. The rare combination of low Ki-67 in HER2-overexpressed cancers (14% of HER2 cancers) showed very slow rate of transition to IDC (only 53.55% of the average rate).

• Luminal B1 phenotype exceeded the average progression rate by 39%, supporting the clinical importance of this phenotype. The Ki-67 subtypes of Luminal B2 (proposed in this study), HER2-overexpressed and triple-negative cancers were not so impressive.

• Low Ki-67 decreased the rate of Luminal B2 tissue progression (27% of Luminal B2 cancers showed 91.9% of the average progression rate).

• A rare combination of low Ki-67 in HER2-overexpressed cancers (14% of HER2 cancers) showed very slow rate of tissue invasion (only 53.55% of the average rate).

• The triple-negative cancers progressed faster than the average rate, despite the Ki-67 value (104.63% for low and 114.27% for high Ki-67).

The rates of tissue invasion for various phenotype combinations of steroid receptor expression are shown in Table 4:

**Table 4:** Relative tumor invasion rates according to the expression of hormonal receptors

**Combined features**			**DCIS**	**IDC**	**Relative rate**	**Rate difference (ER**^**+**^**PgR**^**−**^**ER**^**+**^**PgR**^**+**^**)**
Ductal breast cancer phenotypes of steroid receptor expression	ER^+^PgR^+^		39	799	1.0542	−0.3396
	ER^+^PgR^−^		13	91	0.7146	
	ER^−^PgR^+^		0	11	n/a	
	ER^−^PgR^−^		16	279	1.0016	
	Total		68	1180	1.0000	
Luminal A cancers: steroid receptor phenotype	ER^+^PgR^+^		28	337	0.8824	−0.3785
	ER^+^PgR^−^		6	20	0.5039	
	ER^−^PgR^+^		0	6	n/a	
	Total		34	363	0.8446	
Luminal B1 cancers: steroid receptor phenotype	ER^+^PgR^+^		4	297	1.4849	−0.4554
	ER^+^PgR^−^		2	38	1.0295	
	ER^−^PgR^+^		0	5	n/a	
	Total		6	340	1.3934	
Luminal B2 cancers: steroid receptor phenotype	<=14%	ER^+^PgR^+^	4	45	0.8606	n/a
		ER^+^PgR^−^	0	9	n/a	
	>14%	ER^+^PgR^+^	3	120	1.2756	−0.6718
		ER^+^PgR^−^	5	24	0.6038	
	Total		12	198	0.9832	

In all three tumor types with positive steroid receptors the ER^+^PgR- phenotype showed slower transition rates to the IDC phase than the functional ER^+^PgR^+^ phenotype ( 38% for Luminal A, 46% for Luminal B1 and 67% for Luminal B2 with Ki67 > 14%).

• In all three tumor types with positive steroid receptors the ER^+^PgR^−^ phenotype showed slower tissue progression rates than the functional ER^+^PgR^+^ phenotype (by 38% for Luminal A, 46% for Luminal B1 and 67% for Luminal B2 with Ki67 > 14%), suggesting that if estrogen binding to dysfunctional ERs did not promote PgR expression, it also did not stimulate tissue invasion.

Table 5 shows comparison of calculated relative rates of tissue invasion between patients younger than 55 and older patients:

**Table 5:** Comparison of calculated relative rates of tissue invasion between patients younger than 55 vs. older patients

**Features**	**Age <55 years**			**Age >54 years**			**Rate difference (older-younger)**
	**DCIS**	**IDC**	**Relative rate**	**DCIS**	**IDC**	**Relative rate**	
Luminal A	8	100	0.8944	26	263	0.8277	−0.0667
Luminal B1	2	108	1.3772	4	232	1.4013	0.0241
Luminal B2	4	65	0.9787	8	133	0.9861	0.0074
HER2	4	37	0.7998	6	88	0.9456	0.1458
Triple-negative	2	73	1.2456	4	81	1.0504	−0.1952
**Total**	**18**	**275**	**0.9588**	**44**	**565**	**0.903**	**−0.0558**
ER^+^PgR^+^	14	253	1.0151	25	546	1.0752	0.0601
ER^+^PgR^−^	0	15	n/a	13	76	0.6611	n/a
ER^−^PgR^−^	6	110	1.0198	10	169	0.9914	−0.0284
**Total**	**20**	**378**	**1.0298**	**48**	**791**	**0.9832**	**−0.0466**

The main difference was found in patients with triple-negative tumors that were in younger patients 24% faster than the expected average, while in older patients it was only 5% above the expected value. In HER2-overexpressed tumors, among older patients, the rate of tumor invasion was a just 5% bellow the expected value, but in younger patients the tissue invasion rate was 20% below the expected values. The ER^+^ER- phenotype was 33% slower than expected in older patients and incalculably fast in younger patients with no DCIS found among 15 younger patients.

• The main difference was found in patients with triple-negative tumors whose progression rate exceeded the expected average rate in younger patients by 24%, while in older patients it was only 5% above the expected value.

• In HER2-overexpressed tumors, the rate of tissue invasion among older patients was 5% bellow the expected value, but in younger patients the rate was 20% lower than the expected values.

• All other features, including two steroid receptor phenotypes (ER^+^PgR^+^ and ER^−^PgR^−^) were not found to be dependent on age.

These data suggest that tumor biology in two types of ER negative tumors (HER2-overexpressed and triple-negative tumors) was altered mainly in younger patients. The progression of triple-negative tumors was faster and of HER2-overexpressed tumors slower in younger patients. A possible interpretation regarding HER2-overexpressed tumors is that invasion in these tumors depends on an as yet unidentified mechanism that takes more time to be acquired. In triple-negative tumors, some age dependent mechanism accelerated tissue invasion in younger patients, but it seemed absent after the menopause. Thus, it is possible that we should be looking for another humoral factor, possibly related to ovulatory cycles, although not dependent on the presence of estrogen or progesterone receptors.

## Discussion

This study started from a theoretic paper [1] that addressed several questions regarding tumor invasion into the breast tissue. As shown in previous sections of this paper, assembling a regionally limited case series of breast cancer patients allowed complex questions to be addressed.

Several arguments can be drawn from the presented results. Among the pooled patients’ results from ref. 1, Luminal A was found in near 38% of patients and triple-negative tumors in almost 32% of patients, Luminal B in 20% and HER2-overexpressed in 10% of patients. In our study, the share of triple-negative tumors was smaller (near 13%) while the pooled Luminal A & B1 (analogous to Luminal A in ref. 1) were found in almost 60% of all patients. These differences possibly reflected the improved sensitivity of ER and PgR detection by modern immunohistochemical staining and thus reduced the proportion of triple-negative cancers (from predicted 32% in simulated population to only 12.82% found among the presented cases). This suggests that many tumors among the pooled data from ref. 1., had been classified by the then contemporary immunohistochemical methods as triple-negative, while similar tumors among our patients possibly often met the criteria for Luminal B1 tumors.

The dysfunctional ER^+^PgR^−^ phenotype was more common in Luminal B2 in comparison to the pooled Luminal A&B1 tumors, and also more frequent in Luminal B1 than in Luminal A, suggesting that tumors with HER2 overexpression, or with increased Ki-67 values were in our patients linked to this phenotype with dysfunctional ERs.

On the other hand, the same ER^+^PgR^−^ phenotype among the three tumor types with positive steroid receptors showed slower tissue invasion than the ER^+^PgR^+^ phenotype of the same tumor type (progression rate differences by 38% for Luminal A, 46% for Luminal B1 and 67% for Luminal B2 with Ki67 > 14%). If the presence of functional ERs is so important for tissue invasion in several breast tumor types, a plausible question is whether the efficacy of conventional hormonal therapy is compromised in patients with dysfunctional ERs (those patients with the ER^+^PgR^−^ tumor phenotype).

Tumor progression from DCIS to IDC was found to exceed the average rate by 39% in Luminal B1 tumors, supporting the clinical importance of this tumor type. A rare combination of low Ki-67 in HER2 type cancers showed very slow tissue invasion (only 53.55% of the average rate). Triple-negative cancers showed tissue progression rates above the average, regradless of the Ki-67 value (104.63% for the low and 114.27% for the high Ki-67 tumors). These findings suggest that high Ki-67 values might be used as a surrogate marker of an as yet unrecognized invasion promoting mechanisms in breast tumor types, except among the triple-negative cancers.

When considering age of patients and the relative rate of tissue invasions, breast tumors with the dysfunctional ER^+^PgR^−^ phenotype progressed faster in younger patients and 33% slower than expected in older patients. Triple-negative tumors in younger patients were 24% faster than the expected average, while in HER2-overexpressed tumors, the rate of tumor invasion in younger patients was 20% lower than the expected value. A possible interpretation regarding HER2-overexpressed tumors is that invasion in these tumors depends on an unidentified mechanism that takes more time to be acquired. In triple-negative tumors, an age dependent premenopausal mechanism possibly accelerates tissue invasion without binding to ERs or PgRs (possibly activin/inhibin [16-18] or some other).

## Conclusions

A previously developed theoretic model, from the pooled published breast cancer data [1], suggested that all breast cancer types evolve from DCIS lesions. If so, the more tissue invasive breast cancer types can be recognized by comparing breast tumor type distributions between DCIS lesions and invasive breast cancers, through calculation of the relative rate of tissue invasion.

By using the same approach on a case series from a single institution, the following complex results were obtained:

• Among our patients, the share of DCIS tumors was only 5.45% of all breast cancer cases, probably reflecting late detection of breast tumors in our population.

• The fastest tissue invasion was observed among luminal B1 cancers, supporting the clinical importance of this phenotype and suggesting that the tissue invasion rate of Luminal B1 tumors depends on high Ki-67 value.

• The subgroup of 301 patients with the ER^+^PgR^+^ phenotype of Luminal B1 tumors showed even a higher rate of tumor invasion. Beside that in all three tumor types with positive steroid receptors (Luminal A, B1 and B2), the dysfunctional ERs in the ER^+^PgR^−^ phenotype showed slower rates of tissue invasion. These results suggest that ligand binding to functional breast tumor ERs, beside promoting the PgR expression, possibly also promotes tumor transition to the invasive phase.

• The rare combination of low Ki-67 in HER2-overexpressed cancers (14% of HER2 cancers) showed very slow rate of tissue invasion. This suggests that the phenotype with low Ki-67 in HER2 type cancers might be recognized as a subgroup that longer remain in the DCIS phase at the time of diagnosis than any other cancer types.

The tested model was focused on the very early breast tumor growth phase that leads to tissue invasion, so these results are not necessary important for the subsequent clinical course of invasive ductal cancer. Nevertheless, an investigation into whether the here described subgroups also show differences in subsequent phases of cancer growth or in treatment responses seem warranted.

## Competing interests

The authors declare that they have no competing interests.

## Authors’ contributions

SK participated in the design of the study and performed the statistical analysis, helped to draft the manuscript. Other two authors carried out reevaluation of histological slides and assembled the data base. All authors read and approved the final manuscript.
